# Subterranean Termite Resistance of Polystyrene-Treated Wood from Three Tropical Wood Species

**DOI:** 10.3390/insects7030037

**Published:** 2016-07-21

**Authors:** Yusuf Sudo Hadi, Muh Yusram Massijaya, A. Arinana

**Affiliations:** Bogor Agricultural University, Bogor, Jawa Barat 16680, Indonesia; mymassijaya@yahoo.co.id (M.Y.M.); arinanaiskandaria@yahoo.co.id (A.A.)

**Keywords:** subterranean termite, polystyrene wood, wood weight loss, resistance rating

## Abstract

The objective of this work was to investigate the resistance of three Indonesian wood species to termite attack. Samples from sengon (*Falcataria moluccana*), mangium (*Acacia mangium*), and pine (*Pinus merkusii*) were treated with polystyrene at loading levels of 26.0%, 8.6%, and 7.7%, respectively. Treated and untreated samples were exposed to environmental conditions in the field for 3 months. Untreated specimens of sengon, mangium, and pine had resistance ratings of 3.0, 4.6, and 2.4, respectively, based on a 10-point scale from 0 (no resistance) to 10 (complete or near-complete resistance). Corresponding resistance values of 7.8, 7.2, and 8.2 were determined for specimens treated with polystyrene. Overall weight loss values of 50.3%, 23.3%, and 66.4% were found for untreated sengon, mangium, and pine samples, respectively; for treated samples, the values were 7.6%, 14.4%, and 5.1%, respectively. Based on the findings in this study, overall resistance to termite attack was higher for treated samples compared to untreated samples.

## 1. Introduction

Natural forests in Indonesia cover 93.1 million ha, of which plantation forests account for 4.9 million ha, and the total forested area represents 52% of the total area of the country. Log production in 2013 reached 23.2 million m^3^ of which 84% was from plantation forests that included fast-growing tree species such as sengon (*Falcataria moluccana*), mangium (*Acacia mangium*), pine (*Pinus merkusii*), and gmelina (*Gmelina arborea*) [1]. The logs for this study were cut from young stands, 5 to 10 years old, resulting in timber mostly containing sapwood and juvenile heartwood [2].

Indonesia is a tropical country that has a suitable environment for subterranean termites that attack wooden buildings. Subterranean termite attack of buildings has been reported in all parts of the country, including all districts of Jakarta. By some estimates, the economic loss from termite damage reached at least US $1 billion in 2015 [3].

Compared to heartwood and mature wood, sapwood and juvenile wood have inferior characteristics in terms of physical-mechanical properties and resistance to termite attack. To lengthen the service life of timber, wood preservatives are usually applied, but less toxic methods to increase resistance to biodeterioration and attack by termites have been explored. Alternative techniques of wood preservation have included chemical modification of wood and impregnation of the wood with polymers. In particular, methods have been developed for acetylation of particleboard and fiberboard [4,5] and treatment of wood with polystyrene [6,7], methyl methacrylate [8,9], and furfuryl alcohol [10,11]. Smoked wood [12], smoked glulam [13], and binderless particleboard [14] have also been tested as alternative techniques.

Past studies based on laboratory tests according to Indonesian National Standard [15] showed that jabon wood (*Anthocephalus cadamba*) impregnated with methyl methacrylate has better physical-mechanical properties and resistance against subterranean termite (*Coptotermes curvignathus*) attack than untreated wood. According to other previously published laboratory Indonesian standards, both mindi wood (*Melia azedarach*) with a density of 0.43 g/cm^3^ and sugi wood (*Cryptomeria japonica*) from Japan with a density of 0.34 g/cm^3^ had high levels of resistance to subterranean and dry-wood termite (*Cryptotermes cynocephalus*) attack [16]. A previous study investigated resistance of polystyrene-treated samples of four wood species, namely *Salix alba*, *Alnus glutinosa*, *Populus maximowiczii*, and *Pinus silvestris*, against drywood and subterranean termites indicating that polystyrene wood was much more resistant than untreated wood to the termites [6].

The purpose of the current study was to determine the resistance of polystyrene-impregnated wood from three fast-growing tree species, namely sengon, mangium, and pine, against subterranean termite attack by using a field test method. The hypothesis of this work is that polystyrene-treated wood is more resistant to termite attack than untreated wood because it is more hydrophobic and the voids within the wood are filled with plastic.

## 2. Materials and Methods

### 2.1. Wood Specimen Preparation

**Figure 1:**
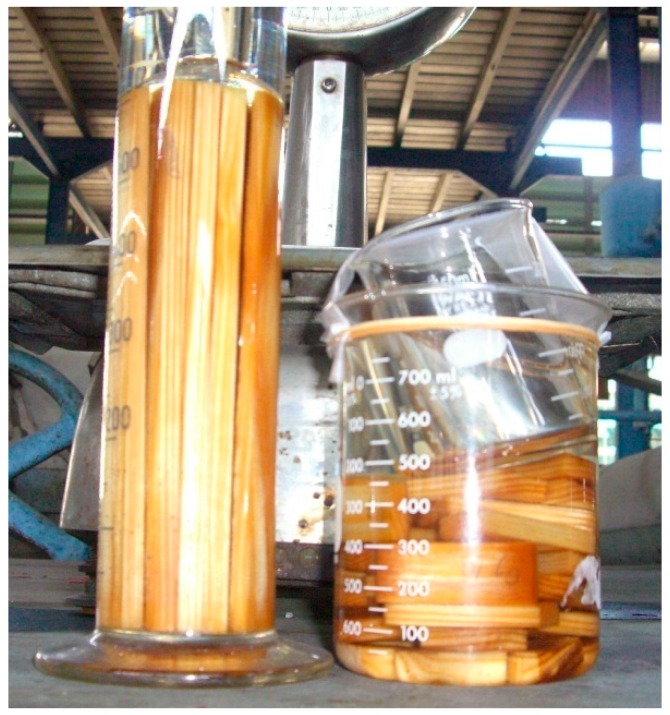
Styrene impregnation of the samples.

Wood from sengon, mangium, and pine trees from Bogor, West Java, Indonesia, was used to determine resistance to subterranean termite attack. The size of test specimens was 1 cm by 2 cm in cross section and 20 cm in the longitudinal direction [8]. The air-dried specimens were weighed prior to polystyrene impregnation. Styrene liquid and catalyst were purchased at Setia Guna Shop (Bogor, Indonesia). Impregnation of the wood with polystyrene was conducted by placing air-dried specimens under vacuum at 600 mm Hg for 30 min, followed by immersion in monomer styrene at 10 kg/cm^2^ for 30 min, after which they were brought to 1 atmospheric pressure (Figure 1). The wood samples were then wrapped with aluminum foil and placed in an oven at 100 °C for 24 h. The aluminum foil was then removed, and each sample was weighed for calculating polymer loading. Five samples of each species were used for both treatments, yielding a total of 30 samples.

### 2.2. In-Ground Testing

**Figure 2:**
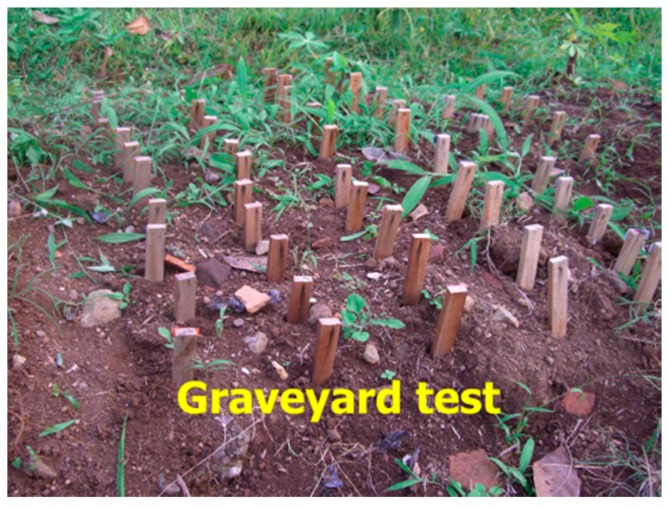
Field test of the specimens.

In-ground testing was conducted at the arboretum of Bogor Agricultural University Campus, Bogor, Indonesia. As shown in Figure 2, each wood sample was pressed into the ground such that only half of its length was aboveground (i.e., graveyard test). The samples were left in place for 3 months. The arboretum is populated with *Macrotermes gilvus* subterranean termites [4]. At the end of the exposure period, the portion of the samples that had been below the ground line was rated to determine the wood resistance against termite attack (Table 1) [5]. The samples were also measured for weight loss (WL) according to the following equation:

WL = (*W*_1_ − *W*_2_)/*W*_1_ × 100%

where *W*_1_ = weight of sample prior to the test (g); and *W*_2_ = weight of sample after the test (g).

### 2.3. Statistical Analysis

The data were analyzed by using a completely randomized block design. The block factor was wood species (i.e., sengon, mangium, and pine), and the treatments were untreated and impregnated with polystyrene. Wood species were block-categorized because all wood species used have low resistance to subterranean termite attack. The linear model could be expressed as:

Y = μ + α + β + ε

whereas: Y = value of observation, μ = average value, α = effect of treatment, β = effect of block, and ε = error.

The significant level was *p* ≤ 0.05.

## 3. Results

The mean values and standard deviations for density and resistance class of untreated wood and the polymer loading, rating of wood resistance against termite attack, and weight loss of untreated and polystyrene wood samples are displayed in Table 2.

The average rating for wood resistance against termite attack for untreated and polystyrene wood samples was 3.3 and 7.7, respectively, and the wood weight loss was 46.7% and 9.0%, respectively.

The analysis of variance for the rating of wood resistance against termite attack is presented in Table 3, and analysis of variance for weight loss is in Table 4. Wood species did not affect the rating of wood resistance against termite attack or wood weight loss, but the effect of treatment on the rating of wood resistance against termite attack and on wood weight loss was significantly different at *p* ≤ 0.01.

## 4. Discussion

Table 2 shows the resistance class of untreated sengon and pine wood as class V, or very poor resistance, and mangium wood as class IV, or poor resistance to subterranean termite attack, according to Indonesian National Standard (SNI) 2006 [18]. The wood specimens were taken from trees that were 5 to 10 years old and predominantly sap wood as indicated by their light color; the heartwood probably had some juvenile wood. The resistance class of wood was not affected by the density [19], as indicated by sengon, with a density of 0.34 g/cm^3^, and pine, with a density of 0.69 g/cm^3^, having the same resistance class V, while mangium, with a density of 0.51 g/cm^3^, had a better resistance class IV. Despite the density of mangium being lower than that of pine, mangium had better resistance. Consequently, it appears that wood resistance to bio-deterioration attack is not related to wood density but the amount and type of toxic extractive content.

For polymer loading, sengon had the highest value followed by mangium and then pine. The order of these values is related to the wood density, from lower to higher values. Lower-density wood was more easily penetrated by styrene monomer, borax, and acetic anhydride because more voids were present compared to higher-density wood [16].

According to the analysis of variance in Table 3, wood species did not affect the rating of wood resistance against termite attack because all three wood species had low resistance to termite attack; however, treatment significantly affected the rating. A higher rating of wood resistance against termite attack indicated that the wood had better resistance. The average rating of polystyrene-treated wood was 7.7, but the rating for untreated wood was 3.3. This difference indicated that polystyrene-treated wood was more resistant than untreated wood against subterranean termite attack. Furthermore, the rating of untreated mangium was higher than sengon and pine because mangium was resistance class IV and both sengon and pine were classified into the lower class, namely class V [17].

Regarding the analysis of variance of wood weight loss as shown in Table 4, the wood species did not affect the wood weight loss, but the treatment had a significant effect. The average weight loss of the three types of untreated wood was 46.7% and that of polystyrene-treated wood samples was 9.0%, indicating that polystyrene-treated wood had much better resistance than untreated wood to subterranean termite attack. This result was in line with Hadi et al. [6,16]. Untreated mangium had the lowest weight loss compared with sengon and pine, and these results were in line with the rating of wood resistance against termite attack.

## 5. Conclusions

The lower-density wood had higher polymer loading of polystyrene compared with higher-density wood. Untreated sengon and pine wood samples had very poor resistance and untreated mangium wood had poor resistance to subterranean termite attack. Polystyrene-treated wood had much higher resistance than untreated samples to subterranean termite attack in a field test.

## Figures and Tables

**Table 1:** Rating system of wood resistance against termite attack.

Rating	Criteria
10	No attack or a few nibbles present.
9	Small tunnel on surface, less than 3% of the cross-sectional area affected at any location.
7	Termite attack affects 10%–25% of the cross-sectional area at any location.
4	Termite attack affects more than 50% of the cross-sectional area at one location, but specimen has not failed.
0	Failure

**Table 2:** Density and resistance class of untreated woods and the polymer loading, wood resistance rating, and wood weight loss of untreated and polystyrene wood samples.

Wood Species	Density (g/cm^3^)	Resistance Class *	Polymer Loading (%)	Wood Resistance Rating		Wood Weight Loss (%)	
				Untreated	Polystyrene	Untreated	Polystyrene
Sengon	0.34 (0.01)	V	26.0 (5.4)	3.0 (3.0) ^a^	7.8 (1.1) ^b^	50.3 (18.8) ^c^	7.6 (2.5) ^d^
Mangium	0.51 (0.01)	IV	8.6 (4.8)	4.6 (1.3) ^a^	7.2 (2.0) ^b^	23.3 (8.1) ^c^	14.4 (7.0) ^d^
Pine	0.69 (0.01)	V	7.7 (2.3)	2.4 (2.2) ^a^	8.2 (1.1) ^b^	66.4 (18.6) ^c^	5.1 (4.3) ^d^

Remarks: Numbers in the parentheses are standard deviation values. * According to Arinana et al. [17]. Values followed by the same letters are not significantly different.

**Table 3:** Analysis of variance of the rating of wood resistance against termite attack.

Effect	SS	df	MS	F	*p* value
Intercept	918.53	1	918.53	233.68	0.000000
Species	2.07	2	1.03	0.26	0.770851
Treatment	145.20	1	145.20	36.94	0.000002
Error	102.20	26	3.93		

Remarks: SS = sum of squares; df = degrees of freedom; MS = mean squares; F = MS at that point divided by MS error; *p* value = significance level.

**Table 4:** Analysis of variance of wood weight loss.

Effect	SS	df	MS	F	*p* value
Intercept	23,302.85	1	23,302.85	89.56418	0.000000
Species	1446.16	2	723.08	2.77915	0.080571
Treatment	10,626.36	1	10,626.36	40.84227	0.000001
Error	6764.69	26	260.18		

Remarks: SS = sum of squares; df = degrees of freedom; MS = mean squares; F = MS at that point divided by MS error; *p* value = significance level.
